# Multi-Cellular Logistics of Collective Cell Migration

**DOI:** 10.1371/journal.pone.0027950

**Published:** 2011-12-21

**Authors:** Masataka Yamao, Honda Naoki, Shin Ishii

**Affiliations:** 1 Graduate School of Information Science, Nara Institute of Science and Technology, Ikoma, Nara, Japan; 2 Graduate School of Informatics, Kyoto University, Uji, Kyoto, Japan; 3 RIKEN Computational Science Research Program, Wako, Saitama, Japan; Université d'Evry val d'Essonne, France

## Abstract

During development, the formation of biological networks (such as organs and neuronal networks) is controlled by multicellular transportation phenomena based on cell migration. In multi-cellular systems, cellular locomotion is restricted by physical interactions with other cells in a crowded space, similar to passengers pushing others out of their way on a packed train. The motion of individual cells is intrinsically stochastic and may be viewed as a type of random walk. However, this walk takes place in a noisy environment because the cell interacts with its randomly moving neighbors. Despite this randomness and complexity, development is highly orchestrated and precisely regulated, following genetic (and even epigenetic) blueprints. Although individual cell migration has long been studied, the manner in which stochasticity affects multi-cellular transportation within the precisely controlled process of development remains largely unknown. To explore the general principles underlying multicellular migration, we focus on the migration of neural crest cells, which migrate collectively and form streams. We introduce a mechanical model of multi-cellular migration. Simulations based on the model show that the migration mode depends on the relative strengths of the noise from migratory and non-migratory cells. Strong noise from migratory cells and weak noise from surrounding cells causes “collective migration,” whereas strong noise from non-migratory cells causes “dispersive migration.” Moreover, our theoretical analyses reveal that migratory cells attract each other over long distances, even without direct mechanical contacts. This effective interaction depends on the stochasticity of the migratory and non-migratory cells. On the basis of these findings, we propose that stochastic behavior at the single-cell level works effectively and precisely to achieve collective migration in multi-cellular systems.

## Introduction

Movements of various cell groups are ubiquitous during development. The extent and speed of migrations must be well-controlled to achieve precise axon placement in the wiring of neuronal networks and to ensure the appropriate morphogenesis of tissues and organs [1]. In this article, we focus on multi-cellular collective migration, which can be observed in the behaviors of cranial neural crest cells during embryonic development, as a model system for understanding how the system-level control of cellular transportation is achieved; such system-level control is called “logistics”. This transportation is accompanied by cell migration that is directed by extra-cellular signaling molecules working as chemo-attractants or repellants. In multi-cellular systems, cellular locomotion is restricted by physical interactions with other cells in a crowded space, similarly to passengers pushing others out of their way on a packed train. The mechanisms underlying multi-cellular logistics in these crowded space remain largely unknown.

At the level of individual cells and neuronal growth cones, migratory behavior is often stochastic rather than deterministic, due largely to the small number of signaling molecules within such cells, which perform biased random walks along chemo-attractant gradients [2], [3]. Nevertheless, the developmental process remains consistent across different embryos, even though the stochastic behavior of individual cells might seem to make precise and consistent control difficult. There must be a homeostasis (stability) mechanism at the multi-cellular systems level that absorbs the stochastic behavior. Also, developmental processes need to be variable enough to construct a variety of biological patterns starting from a single fertilized egg cell, while being stable enough to maintain the consistency of the patterns; this requirement is a typical plasticity-stability dilemma [4]. Therefore, the relationship between microscopic properties of individual cell migration and macroscopic multi-cellular migration patterns needs to be clarified.

Multi-cellular migration employs various modes of transportation, depending on the cell type and the developmental stage. These modes can be classified into two main categories, individual and collective migration [5]. Individual migration is dispersive and enables cells to cover a local area, as can be seen in immune cell trafficking [6]. Collective migration consists of multi-cellular units and is used mainly to build complex tissues. Typically, neural crest cells migrate together by a forming “stream” [7], and neural precursor cells sometimes migrate along a single dimension in a “chain”-like manner [8]. Understanding how these modes of migration emerge is important for understanding the mechanisms of multi-cellular development.

It has recently been shown that pattern modes can be experimentally inter-converted by manipulating the expression of proteins involved in cellular mechanics; up-regulating a cell adhesion molecule (CAM) in individually migratory cells leads to collective migration [9], whereas down-regulating a CAM in collectively migrating cohorts leads to individual migration [10], [11]. These observations suggest that the various transport pattern modes are not achieved simply by system-specific molecular regulations. In addition, it has been suggested that the pattern modes can be controlled through altering physical parameters in cell migration such as driving force, cellular stiffness, and the randomness of the migration [12]. However,the mechanisms by which microscopic mechanical parameters at the level of single cells contribute to the macroscopic pattern of multi-cellular migration remains elusive.

In this paper, we studied the multi-cellular logistics of biological systems with a special emphasis on collective migration in a crowded environment. To this end, we focused on “neural crest migration”, because even without the guidance of extra-cellular signals, neural crest cells collectively migrate from rhombomeres to branchial arches along stream [13]. We constructed a bio-physical model of a multicellular system in which cells migrate through crowded cell population using their chemotactic abilities. Note that neural crest cell migration is driven by both chemotactic abilities and population pressure due to proliferation[14], [15]. In our study, we particularly examined cell migration phenomena in a crowded situation. We then performed a computer simulation, which led us to hypothesize that migratory cells exploit the stochasticity within multi-cellular systems to collectively and efficiently migrate using an autonomously emerging stream. Our theoretical analysis could shed light on the mechanisms that govern various migration pattern modes. Moreover, we discuss the properties of multi-cellular logistics on the basis of our simulation.

## Results

### Model of multi-cellular migration

**Figure 1:**
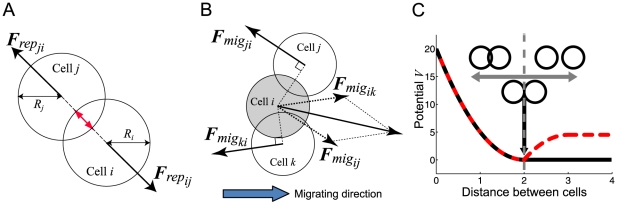
Model for simulation. (A) When two neighboring cells indicated by the white circles overlap, the repulsive force (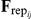 and 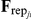) is proportional to the degree of overlap, as indicated by the red arrow. (B) The migrating cell (indicated by a gray circle) is assumed to be attracted by a chemo-attractant gradient. Its driving force (the sum of 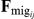 and 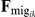) is generated at points of contact with other cells, whereas reactive forces (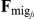 and 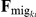) are applied in the direction opposite to that of the attractant gradient regardless of the cell type (migratory or non-migratory). (C) The repulsive forces when the cells contact and the attractive adhesive forces when the cells are close are given by the gradient of the potential 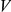. The black and dashed red lines indicate the potential 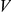 for Equations (1) and (8), respectively. The black arrow indicates a steady-state point at which the two cells just contact.

To examine the general properties of multi-cellular migration, we developed a bio-physical model that includes the essential characteristics of the mechanical nature of general multi-cellular systems. This model multi-cellular system consists of a number of mechanically interacting cells (Figure 1). Each cell is represented as a two-dimensional disk with a static body. The simple multi-cellular migration model consisted of three forces: (1) the repulsive force between cells, (2) the driving force of migratory cells accompanied by reaction forces of neighboring cells via adhesion, and (3) the stochastic forces involved in a random walk. Assuming that the viscosity of the cellular environment is sufficiently high, the inertia can be ignored, and the viscous drag force is exactly balanced between these forces. Thus, the dynamics of the cellular positions 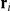 are described as follows: 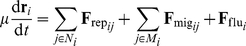
(1)

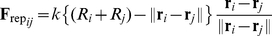
(2)

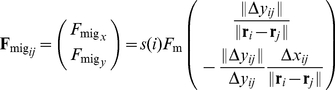
(3)

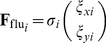
(4)

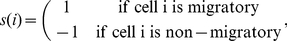
(5)where 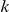 is the Young's modulus, 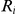 is the radius of cell 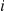, 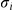 is the fluctuation intensity of cell 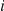, 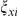 and 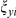 are independent random functions of time with mean zero, 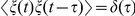 is the autocorrelation function, 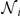 is the index set of all cells contacting cell 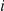, 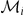 is the index set of the other type of cells contacting cell 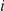, 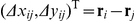, and 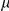 is the viscous modulus. Equation (2) holds only when 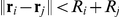. Otherwise 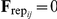.

The repulsive force is induced by compressive deformation of the cells due to the elasticity of their cytoskeletons and plasma membranes [16]. Even though the cells are modeled as static bodies, we implicitly address this morphological compression by introducing a repulsive force; when two adjacent cells overlap (contact) each other, a repulsive force 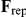 is generated between them (Figure 1A). This repulsive force is directed so as to separate the contacting cells, and its strength decreases in proportion to the distance between them.

The driving force is generated when a migratory cell adheres through a pseudopod, which is an actin rich peripheral structure that promotes cellular motility [17]. To model cell migration, we consider two types of cells: migratory and non-migratory. Migratory cells are assumed to have the chemotactic ability to be attracted by extra-cellular signals and thereby travel along their gradient. We do not focus on the molecular mechanisms sensing the gradient here and instead just set the migration direction. Because a migratory cell adheres to all contacting non-migratory cells so as to use them as footholds for migration, a driving force 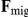 is tangentially generated between the cells along the direction of the extra-cellular attractant gradient. Consistent with the principles of action and reaction, the non-migratory cells experience a force opposing the force driving the migration (Figure 1B), which causes the non-migratory cells to be pulled backward and the migratory cell to proceed forward. Note that when two migratory cells contact, the action (driving force) generated by one migratory cell is cancelled by the reaction from the other migratory cell's driving force, and neither migratory cell is propelled forward. These assumptions are implemented by introducing simple geometrical rules: 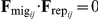, 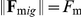, and 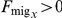. These rules lead to Equation (3).

The stochastic forces 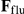 are autonomously generated by the inherent intracellular dynamics [2], [3]. In the model, both the migratory and non-migratory cells are assumed to spontaneously display random movements even if they do not experience the above-mentioned forces. We modeled this randomness as a Gaussian random function.

To reduce the number of free parameters in the model equations of this study, we applied a non-dimensionalization technique to the original bio-physical Equations (1*–*5). We then have only three non-dimensionalized free parameters, which correspond to Young's modulus, the noise intensity of the migratory cells, and the noise intensity of the non-migratory cells (See the Materials and Methods section). Here, the typical value of the non-dimensionalized Young's modulus, 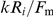, becomes approximately 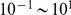 by introducing typical values for Young's modulus, the cell radius, and the migration force into the original bio-physical model. These values are 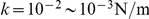 [16], [18], 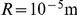, and 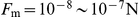 [18], respectively. A typical value for the non-dimensionalized noise intensity, 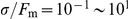, is also derived from the typical value of the noise intensity in the bio-physical model, 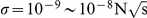 [18 19]. Accordingly, we primarily use the parameter values 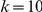, and 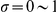 as biologically plausible values in this study.

**Figure 2:**
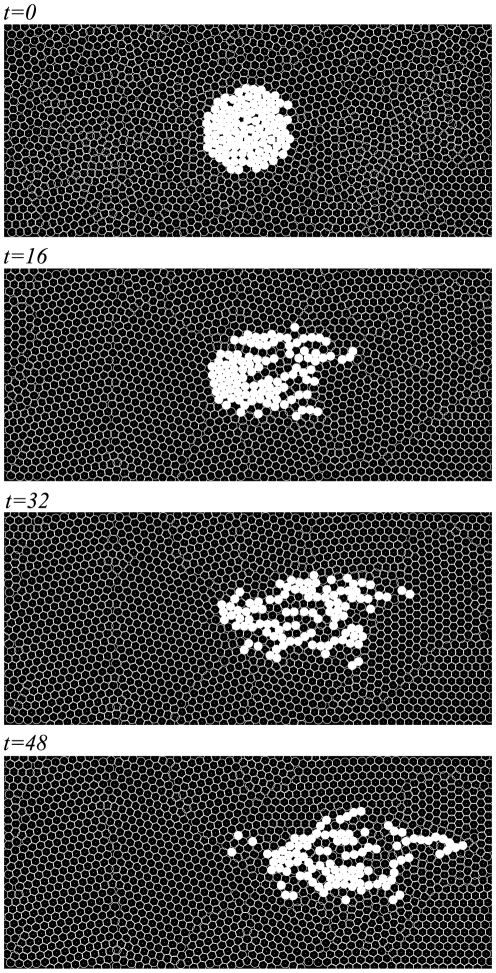
Snapshots of a single simulation series of multi-cellular migration. The white and black disks indicate migratory and non-migratory cells, respectively. The migratory cells are initially (at 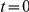) distributed as a cluster (upper panel) and then migrate rightward progressively at 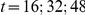 (the lower three panels). The fluctuation intensities for the migratory and non-migratory cells are set to 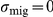 and 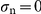, respectively.

For the sake of ignoring boundary effects, all the cells are assumed to be packed into a two-dimensional rectangular space with boundaries that are connected to form a torus structure. At the beginning of each simulation run, 100 migratory cells were distributed around the center and then transferred rightward (Figure 2).

### Effect of single cellular stochasticity on multi-cellular transportation

**Figure 3:**
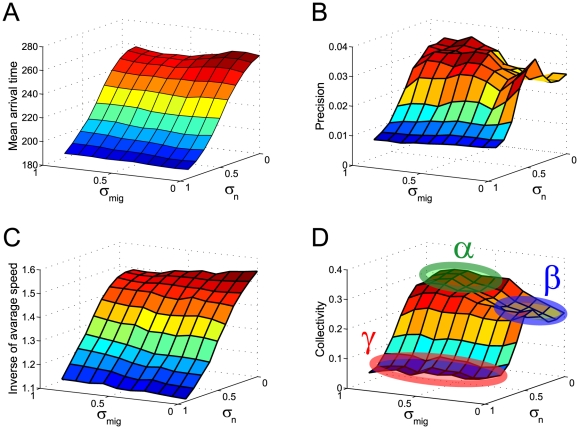
The cellular migration characteristics of multi-cellular systems depend on the relative fluctuation levels of the migratory (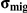) and non-migratory (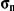) cells. The average time for a migratory cell to reach its target position 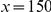 (A) and the inverse of the variance in the position of the migratory cell after arriving at the position (B) are plotted. The inverses of the mean velocity (C) and collectivity (D) of the migratory cells are plotted at a quasi-steady state after the initial transient phase. Here, collectivity is defined by Equation (6), with 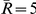 and 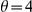. In (D), there are three typical collectivity patterns, signified by 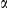, 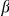, and 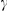.

**Figure 4:**
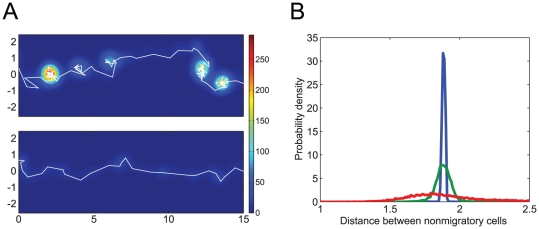
The mesoscopic behaviors of migratory and non-migratory cells. (A) Simulations were performed with a single migratory cell surrounded by non-migratory cells. The white lines indicate sample two-dimensional trajectories of the migratory cells with parameter values corresponding to the points indicated by 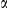 and 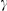 in Figure 3D. The color contour indicates the density of the migratory cells as calculated by the kernel density estimation using Gaussian kernel functions. (B) Each line shows the distribution of distances between neighboring non-migratory cells when simulating a multi-cellular system that has only non-migratory cells and no migratory cell. The green, blue, and red lines correspond to the cases that have parameter values in the three regions, 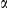, 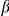, and 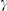 in Figure 3D.

Because migratory cells in real developmental situations target a specific location and then differentiate within a specific developmental stage, their transportability and transportation accuracy are determinants of their eventual configuration. Therefore, these characteristics were examined in our simulation. First, transportability was examined by varying the fluctuation intensities of the migratory and non-migratory cells, 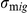 and 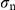. We defined transportability here as the average time required for the migratory cells to reach a specific goal position (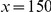 of the rectangular space). The transportation speed (the inverse of the mean arrival time) was found to increase as the fluctuations of the non-migratory cells strengthened (Figure 3A). This noise-induced transportability can be understood on the basis of the following mesoscopic analysis (Figure 4). With relatively small fluctuations in the non-migratory cells, a migratory cell slowly migrates in a hopping manner by pushing other cells out of its way (Figure 4A, upper panel). When the non-migratory cells have large fluctuation, cell migration is smooth and rapid (Figure 4A, lower panel) because the large fluctuation cause the distances between adjacent non-migratory cells to vary significantly, which enables the migratory cell to move easily between them (Figure 4B).

Second, we evaluated transportation accuracy, which is defined as the inverse of the variance in the position of a migratory cell that has arrived at the target position (Figure 3B). Transportation accuracy was found to be high, especially when the migratory cells fluctuated significantly and the non-migratory cells did not. It is interesting that significant fluctuation in the non-migratory cells naturally led to dispersed migration, whereas significant fluctuation in the migratory cells led to increase accuracy. Figures 3A and 3B suggest that transportation in the multi-cellular system exhibits diverse transportability and accuracy patterns depending on the fluctuation levels of the migratory and non-migratory cells and that there is tradeoff between transportability and accuracy.

**Figure 5:**
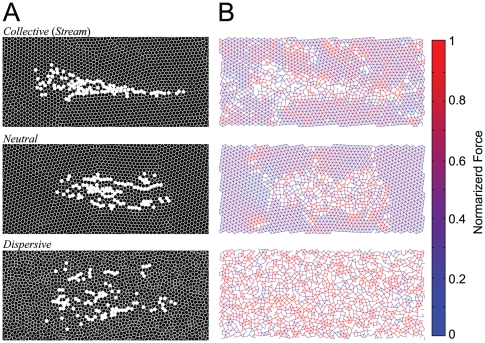
Snapshots of migration patterns, cell contacts and the migratory cell population. The upper, middle, and lower panels show the migratory patterns corresponding to the parameters indicated by 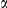, 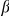, and 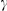 in Figure 3D. (A) The migration patterns at a specific point in time are shown. The white and black circles indicate migratory and non-migratory cells, respectively. (B) The cell contacts are shown at the same time point as in (A). The links depict contacts between cells that interact by repulsive elastic forces (Figure 1A), the strengths of which are indicated by their brightness (for red), or darkness (for blue).

Because the migratory cells are assumed to be initially aggregated (top panel in Figure 2) and then move to the target position, the cell-migration properties shown in Figures 3A and 3B include transient effects. To examine the population behavior that is independent of such transient effects, we performed additional long-term simulations in which the population behavior reaches a quasi-steady state. We then characterized the steady-state logistics in terms of the inverse of the mean velocity (Figure 3C) and the collectivity of the migratory cells (Figure 3D). Here, collectivity was quantified as 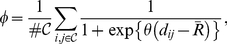
(6)where 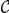, 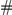, 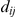, 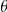, and 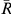 denote the index set of the migratory cells, the operator that counts the number of elements of a set, the distance between two migratory cells 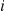 and 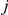, the steepness of the sigmoidal fitting, and the arbitrary radius of focused regions centered on each migratory cell, respectively. This equation approximates the average number of migratory cells around themselves within the radius of 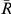. Figures 3C and 3D are roughly comparable to Figures 3A and 3B, respectively. Figure 3D shows three characteristic parameter regions (indicated by 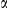, 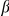, and 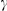) wherein high, intermediate, and low collective patterns, respectively, are realized. Figure 5A shows the characteristic migratory patterns realized by different combinations of 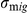 and 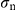 in each of the three parameter regions in Figure 3D. First, when the migratory cells fluctuate significantly and non-migratory cells do not (point 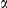 in Figure 3D), the migratory cells collectively converge into one large cellular stream (“ *collective migration*”) (upper panel in Figure 5A and Movie S1); this behavior is similar to that of neural crest cells migrating from rhombomeres to branchial arches [7]. Second, at the point 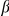 in Figure 3D where the fluctuations of all the cells are weak, the dispersion of the migratory cells does not change significantly during their migration (“ *neutral migration*”) (middle panel in Figure 5A and Movie S2). Third, when the non-migratory cells fluctuate significantly (point 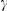 in Figure 3D), the migratory cells disperse rapidly, and each migratory cell comes to migrate individually (“ *dispersive migration*”), regardless of the fluctuation of the migratory cells (lower panels in Figure 5A and Movie S3). These simulation results show that even though the migratory cells are represented as mechanically passive disks lacking information processing by intra-cellular signal transduction, this multi-cellular system has the potential to exhibit cellular migration and to show various migration patterns that are induced by both intrinsic and environmental fluctuations.

### Interaction between migratory cells

**Figure 6:**
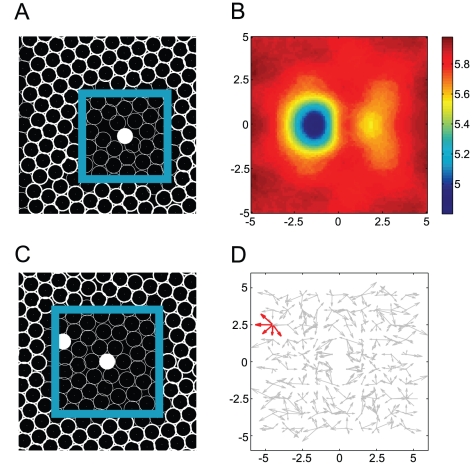
An illustration of the method for estimating cell density and the effective potential field around a migratory cell. The simulations were performed after placing a single migratory cell (A) or two migratory cells (C) to be surrounded by non-migratory cells. The white and black circles indicate migratory and non-migratory cells, respectively. (B) The average density of the non-migratory cells was estimated relative to the position of the migratory cell at the origin (i.e., (A)). This density is estimated by kernel density estimation with Gaussian kernel functions with variances equal to the cellular radius. The square region shown in this panel corresponds to the cyan square in (B). (D) The sample-based velocity vector field. We performed a short-term (0.5 sec.) simulation after placing a migratory cell on each grid point, and the vector differences of each migratory cell in its position are displayed at the each grid points. The square region shown in this panel corresponds to the cyan square in (C).

**Figure 7:**
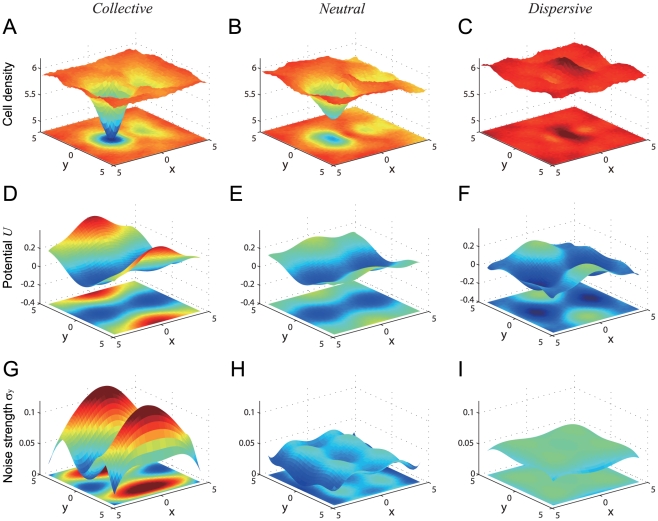
Simulation-based determination of effective cellular interaction. (A,D,G), (B,E,H), and (C,F,I) show the collective, neutral, and dispersive migration modes corresponding to the parameters indicated by 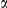, 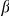, and 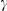 in Figure 3D, respectively. The 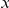 and 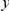 axes indicate spatial coordinates relative to a migratory cell in Figures 6A and C. (A-C) The average cellular density is estimated by the same method as in Figure 6B. The potential landscape (D-F) and effective noise intensity along the 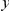 axis (G-I) are estimated using a least-square regression for polynomial functions. Please see the Materials and Methods section.

How is collective migration (a macroscopic behavior) realized when cells mechanically interact with neighboring cells? In many migration patterns, migratory cells were found to follow other migratory cells (Movie S1). Migratory cells can easily invade non-migratory cells because a preceding migratory cell produces some null space in its wake, implying that a migratory cell affects the positional configuration of the surrounding cells. To visualize such configurations, we performed a simulation run with a single migratory cell surrounded by non-migratory cells (Figure 6A), and we evaluated the average density of the non-migratory cells around the single migratory cell (Figure 6B), which reflects the spatial profile of the pressure caused by repulsive interactions. With parameter values in the *collective* migration mode, the average density of non-migratory cells was much lower behind the migratory cell than in other location (Figure 7A). This low density region is similar to the null space and can be interpreted as a low-pressure region where cells easily invade due to their morphological deformation. The average density of non-migratory cells was slightly lower behind the migratory cell under *neutral* migration parameter values (Figure 7B), and it was almost constant with *dispersive* values (Figure 7C). These results revealed that the fluctuations of migratory cells help to form a null space that induces other migratory cells to follow in their wake, whereas the fluctuations of non-migratory cells erase the wakes of the migrating cells.

Our analysis above indicates that as long as migratory cells are close to one another, they stay close due to the effective attraction induced by the null space, and this effect contributes to the stability of the collective migration. However, there is still a missing link: how do separate migratory cells aggregate? Two possibilities are conceivable. First, they may randomly migrate, by chance encounter each other, and then follow one another. Alternatively, they may be actively attracted even in the absence of direct contact, through long-distance effects resulting from direct interactions with non-migratory cells. To examine which possibility is more plausible, we determined the degree of effective interaction between separated migratory cells.

For simplicity, we considered a two-body interaction between two migratory cells under the assumption that their movement follows Brownian motion under an effective potential field. If there is an effective potential field 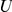, the Brownian dynamics of the migratory cells can be expressed by a stochastic differential equation: 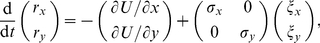
(7)where 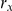 and 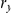 denote relative location coordinates of the two migratory cells, 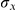 and 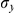 are the effective noise intensities along the horizontal and vertical axes of the rectangular space, 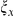 and 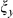 are independent random functions of time with mean zero, and 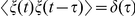 is the autocorrelation function. Because this dynamics is “effective”, we identified the effective potential 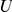 and noise intensities, 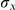 and 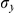, using simulation; we simulated a system that includes only two migratory cells (Figure 6C) and then sampled the velocity vector field 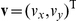 as a function of the relative coordinates between the two cells (Figure 6D) (see the Materials and Methods section).

Figures 7D-F (and 7G-I) show the estimated effective potential 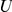 (and the noise intensities 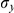) for the collective, neutral, and dispersive migration modes, respectively. In all cases, the potential landscapes have one saddle node and two stable points. This equilibrium point structure implies a situation in which two migratory cells are effectively attracted to each other. In the case of collective migration, the potential gradient is steeper, and 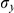 is higher on both sides of the migratory cell. The migratory cells each appear to search for the stable point by utilizing a higher 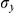 and a steeper gradient; once they reach the stable point, it is difficult for them to escape from it because of the lower 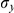. In neutral migration, the potential gradient is gentle and the fluctuation intensity is low over the area around the migratory cells. In the case of dispersive migration, however, the potential gradient is gentle but disturbed by strong noise, suggesting that the migratory cells can easily escape from the stable points and move away from each other.

### Other properties of multi-cellular logistics

**Figure 8:**
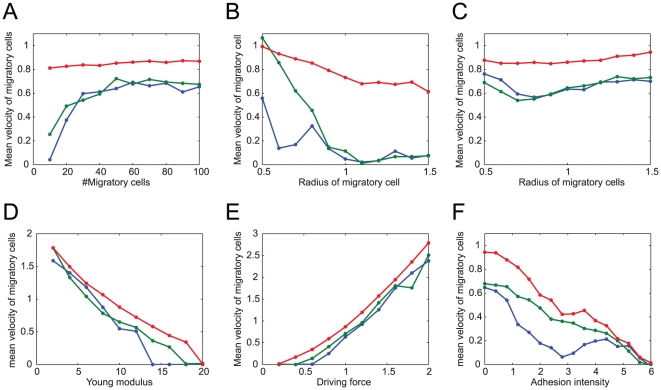
Dependence of transportability on the physical parameters of cells. The green, blue, and red lines represent collective, neutral, and dispersive migrations corresponding to the parameters indicated by 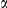, 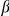, and 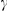 in Figure 3D, respectively. The average migratory cell speeds are plotted according to various values for population size (A), migratory cells radius (B, C), migration driving force (D), and Young's modulus for all cells (E). In (F), an additional attractive force from cell adhesion is included in the model by using Equation (8) (see also the text) instead of Equation (1), and its intensity 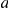 is varied. In (A), (C), (D), (E), and (F), the setting of the migratory and non-migratory cells is similar to that in Figure 2, with the addition of the attractive force in (F), whereas in (B), there is only a single migratory cell surrounded by non-migratory cells.

We next investigated the parameter dependence of transportability (Figure 8). For the collective and neutral migration modes, we found that an increase in the number of migratory cells increased the speed of the collective migration in a saturating manner (Figure 8A, blue and green lines), which is consistent with experimental observations [20]. This population-based transportability likely occurred because migratory cells broke their contacts with the non-migratory cells (Figure 5B, upper and middle), leading to energetically efficient migration. By contrast, the velocity of dispersive migration was unaffected by population size (Figure 8A, red line) because the noisy environment broke the contacts between the non-migratory cells, enabling the migratory cells not for necessitate population-based migration (Figure 5B, lower).

Because cell size changes drastically between different developmental stages, we next examined how the size of a migratory cell affects its transportability. When a cell migrated alone, its migration speed was found to decrease as its cellular radius increased, regardless of the migration mode (Figure 8B). Note that the migration speed is highest in the dispersive migration mode (Figure 5B, bottom). With population-based migration, however, cell size was found to affect migration in a complicated fashion (Figure 8C). For the collective and neutral migration modes, there are two characteristic phases; as the size increases, the migration speed first decrease and then increases when the size exceeds a certain threshold. The first phase exhibits behaviors similar to those observed when there is only a single migratory cell (Figure 8B). The second phase could be attributed to a population effect, through which a large migratory cell produces a large null space in its wake and is effectively followed by other migratory cells.

During cell migration, extra- and intra-cellular signaling actively control cellular stiffness, force generation, and adhesion via regulating cytoskeletal components such as actin filaments and microtubules. In our simulation, the migration speed was found to decrease as the cells stiffen (Figure 8D), because stiff non-migratory cells require a larger driving force to allow the migratory cells to invade. When the cells are stiffer, i.e., when the Young's modulus exceeds 18, the multi-cellular system behaves completely differently; the migratory cells do not proceed any further.

**Figure 9:**
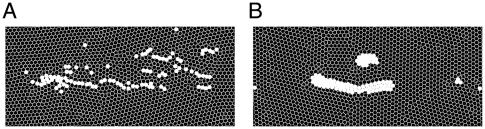
Dependence of migration mode on the driving force and cellular adhesion. (A) Collective migration collapsed when the driving force was too strong. The parameters values are identical to those in the top panel of Figure 5A, i.e., to the collective migration parameters with the driving force (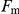) doubled. (B) Chain- or cluster-like migration was induced by introducing an attractive force due to cellular adhesion, i.e., Equation (9). The attractive force intensity is 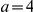, the effective distance of the attractive force is 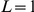, and the other parameter values are those of the characteristic point 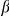 in Figure 3D.

A larger driving force was found to trivially increase the migration speed (Figure 8E); however, it also eliminates collective migration (Figure 9A) because a powerful migratory cell easily pushes the non-migratory cells out of its way and makes the effect of the null space less important in migration. This interpretation also suggests that there is a trade-off between migration velocity and collectivity, as seen in Figure 3.

The effects of adhesion molecules are implicitly reflected in the model by the action-reaction force between contacting cells. However, adhesion molecules play other roles in attracting cells. To examine whether the adhesion force does or does not affect multi-cellular migration, we further extend the model to include an attractive adhesion force between neighboring migratory cells that are located within a distance of 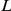: 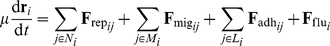
(8)

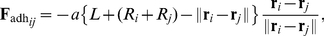
(9)where 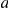 is the intensity of the attractive force and 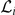 is the set of migratory cells that satisfy the condition, 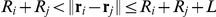. In this extension, the strength of the attractive force is designed to increase as the cells become closer (as in Equation (9)) because the number of binding adhesion molecules, which generates the attractive force, increases as the cells become closer. To see the difference between Equations (1) and (8), we depict the potential 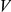 for those equations; the repulsive and attractive forces depend on the potential gradient according to 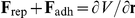 (Figure 1C). In the previous equation, the migratory cells are only repelled when they come in contact (black line in Figure 1C), whereas in the extended model that employs this new equation, the migratory cells are attracted when they become close (red line in Figure 1C). The typical orders of 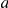 and 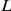 are approximately 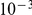
N/m and 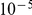
m [18], [21], respectively, and their non-dimensionalized values become 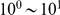 and 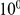, respectively.

We found that the attractive force inhibits the migrating speed (Figure 8F). Interestingly, the additional attractive force changed the neutral migration mode to another mode characterized by forming a cluster or chain (Figure 9B), as experimentally observed in cancer cell migration [10]. This result implies that cellular adhesion can be involved in generating migration patterns accompanied by cluster and chain-like behaviors.

## Discussion

During multi-cellular development, in addition to intra-cellular biochemical features, mechanical cellular features evoked by direct physical interactions with neighboring cells become dominant. By simulating multi-cellular migration using simple mechanical cells, we have shown that microscopic stochasticity plays a significant role in the emergence of population migration patterns and their logistics.

Our model can explain the collective migration of neural crest cells, which occurs through the autonomous formation of a stream (Figure 5A, upper panel). Stream formation has been hypothesized to be extra-cellularly regulated by repulsive cue molecules [22]. Recently, however, it has been reported that down-regulation of the repulsive cue molecule neurophilin-1 does not affect the collective migration of neural crest cells [7]. Our model has indicated that the combination of strongly fluctuating migratory cells and weakly fluctuating non-migratory cells lead to collective migration with autonomously stream formation (point 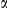 in Figure 3D). Although the mechanisms by which chemotactic cells manage to suppress the intrinsic stochasticity of signal transduction have been previously discussed [3], the ways in which multicellular functions are implemented in the context of the stochastic migration of individual cells have not been examined.

Several theoretical models have been proposed for multi-cellular migration [23], [24]. The dynamics of the cell population have often been modeled by reaction-diffusion systems [25]. Although approaches based on reaction-diffusion systems likely ignore the detailed dynamics at the single-cell level, they are still useful for providing insight into macroscopic mechanisms. A previous theoretical study addressed the migration of neural crest cells in the intestine, thereby highlighting the biological significance of cell proliferation [14]. Proliferative activity was also found in the cranial neural crest cells that we addressed in this study [15]. Therefore developing a model that includes this proliferation is an important step towards reproducing multi-cellular migration more realistically. Proliferation was easily introduced into our model by adding a new cell near the existing cell, as in an existing computational model [26]. An alternative approach is the phase-field model, in which cellular morphological changes are represented by partial-differential equations that are derived by minimizing certain energy functions; this model has been applied to both cellular migration [27] and proliferation [28]. Because the phase-field model can accommodate flexible morphological changes at the single cell level, it is suitable for simulations of population migration with proliferation.

In our model, multi-cellular migration was simply modeled using three forces: (1) the repulsive force between cells, (2) the driving force of migratory cells accompanied by the reactive forces of neighboring cells via adhesion, and (3) the stochastic forces involved in a random walk. These forces are biologically reasonable for the following reason. The repulsive force is induced by the compressive deformation of the cellular morphology and results from the elasticity of the cytoskeleton and plasma membrane [16]. The driving force is generated when a migratory cell extends a pseudopod that adheres to another cell [17]. The stochastic forces are autonomously generated by inherent intracellular dynamics [2], [3]. Although we ignored the complex rheological properties of such structures [8], the minimal model we adopted is still useful for understanding the system-level properties of multi-cellular migration. We propose that our simulation and method of identifying cellular interactions can be applied to other simple developmental systems. Such systems include fibroblasts and neural precursor cells (which sometimes migrate one-dimensionally in a “chain”-like manner) [8], drosophila border cells during oogenesis, and the zebrafish neurons during the development of lateral lines that migrate as a “cluster”.

Nevertheless, the *in vivo* mechanisms of cellular migration must be more complicated than those assumed in this study. In reality, a chemotactic cell shows morphological changes, such as extensions of special structures called filopodia and lamellipodia, through which the cytoskeletal network regulates cell motility [29]. When migratory neural crest cells collide, their migration transiently stops, and their morphological polarities are reorganized, a process known as “contact inhibition” [30]. Furthermore, proliferation and differentiation play important roles in the development of neural precursor cells and neural crest cells, and these behaviors are controlled by an extracellular Wnt signaling gradient. In the neural tube, proliferation and differentiation are induced by high and low levels, respectively, of the Wnt signal molecule, and the Wnt gradient thereby regulates pattern formation [31]. Neural crest cell requires the Wnt signal for their induction in the dorsal neural tube [32], their delamination from the dorsal neural tube [33], and to acquire motility [33]. Such effects are important for understanding the development of complete multi-cellular systems; because our current study focuses on collective cellular migration, studying these effects remain as a future objective.

The collective behavior of populations of self-propelled particles has been studied in the context of many biological and social phenomena, such as schools of fish, flocks of birds [34], ant trails [35], [36], cars in traffic jams [37], and cellular slime molds [18]. In such systems, individual particles actively process external information provided by other particles, and this process induces collective behavior. By contrast, our study obtained non-trivial simulation results in which cells collectively migrate solely in response to crowding effects and in the absence of active information processing. Therefore, our work is the first to present a feasible model for the emergent collective behaviors displayed by multi-cellular systems in crowded situations. Moreover, our model may provide general insight into the universal mechanisms underlying a large class of complex systems that consist of crowded self-propelled particles, such as pedestrian flow [37], solution of charged colloids in electric fields [38], and other multi-cellular developmental processes [39].

## Materials and Methods

### Non-dimensionalization

The original bio-physical model given by Equations (1*–*5) is non-dimensionalized by setting a common cellular radius for all of the cells, i.e., 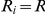 for all 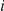, as follows: 
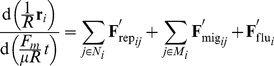
(10)

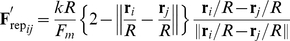
(11)

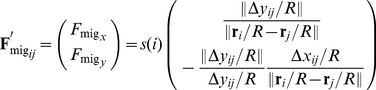
(12)

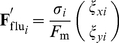
(13)

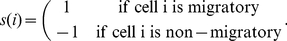
(14)


We have defined the dimension-less variables as 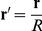, 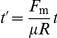, 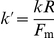, and 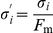 here. After this non-dimensionalization, the free parameters of the model are reduced to 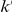 and 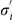, implying that the noise intensity differs between the migratory and non-migratory cells.

The adhesive force in the model is also non-dimensionalized as follows: 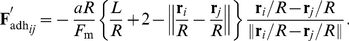
(15)


Then, we set 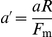 and 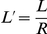.

### Effective interaction identification

We proposed a method for identifying the effective potential 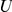, which is defined in Equation (7). Because the non-parametric (sample-based) estimation of the velocity vector field, 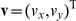, can be rough due to the lack of continuous constraint, the potential was modeled as a continuous parametric polynomial function: 
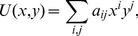
(16)where 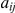 is the coefficient of 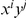 and 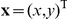 denotes the relative coordinates in two-dimensional space. Because 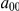 does not affect the dynamics (Equation (7)), it was simply set to 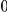. The relative velocity of the migrating cell is then re-expressed by 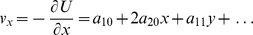
(17)

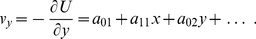
(18)


These coefficients were estimated by a least-squares regression on the basis of the vector field sampled by the simulations (Figure 6D): 
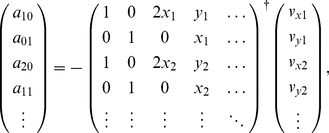
(19)where 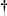 denotes the Moore-Penrose pseudo-inverse. Because the effective noise intensity along the vertical axis is important for collectivity, 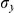 was defined as the mean-squared error between the sampled velocity 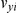 at 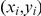 and its expected value, 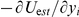, i.e., 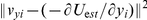. To smoothly estimate the position-based effective variance, we again used a polynomial fitting: 
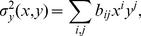
(20)the coefficients of which, 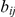, were identified using a least-squares regression on the simulation samples: 
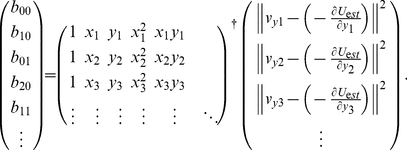
(21)


## Supporting Information

Movie S1 **Simulation of “** ***collective migration*** **” in multi-cellular system.** This movie corresponds to upper panel in Figure 5A.
(MP4)

Movie S2 **Simulation of “** ***neutral migration*** **” in multi-cellular system.** This movie corresponds to middle panel in Figure 5A.
(MP4)

Movie S3 **Simulation of “** ***dispersive migration*** **” in multi-cellular system.** This movie corresponds to lower panel in Figure 5A.
(MP4)
